# Dr. F. T. Paine’s Discoveries in Electro-Therapeutics

**Published:** 1889-10

**Authors:** 


### EDITORIAL DEPARTMENT.

F. E. DANIEL, M. D., EDITOR AND PUBLISHER.

THIS JOURNAL, by unanimous vote of the members, was made the Official Organ of the Austin District Medical Society.

COLLABORATORS:

*Dr. R. M. Swearingen, Austin.*
*Prof. B. E. Hadra, M. D., Galveston.*
*Prof. Geo. Cupples, M. D., San Antonio.*
*Dr. T. C. Osborn, Cleburne, Texas.*
*Dr E. J. Doering, Chicago.*
*Dr. E. J. Beall, Fort Worth.*
*Dr Odo Betz, Germany.*
*Dr. J. W. McLaughlin, Austin.*
*Dr. T. J. Tyner, Austin.*
*Prof. J. F. Y. Paine, M. D., Galveston.*
*Dr. R. H. L. Bibb, Mexico.*
*Dr. T. J. Bennett. Austin.*
*Dr. Bat Smith, Wharton.*
*Dr. E. Meierhof, New York.*
*Prof. W. B. Rogers, M. D., Memphis, Tenn.*

### DR. F. T. PAINE'S DISCOVERIES IN ELECTRO-THERAPEUTICS.

Notwithstanding what was said in reviewing the Transactions, about discriminating in criticising the papers contained therein, we cannot resist the inclination to call attention to the paper by Dr. F. T. Paine, on account of certain most remarkable statements therein made. The importance of the subject prompts us to do so; and Dr. Paine's age and position in the profession will, we hope, justify us in making an exception of his paper. We trust we may be permitted to do so without exciting the jealousy or animosity of any of the gentlemen who have equally as good papers in the volume. If further experiment and observation shall confirm the correctness of Dr. Paine's deductions; if others find their experience with electricity tally with that of this patient investigator, it will mark a wonderful advance in therapeutics; chloroform in labor will be superceded by an agent less dangerous and more certain. Dr. Paine says:

"When we commenced the use of electricity [in obstetrics], we were ignorant of the effect to be expected from it; whether it was to act as an oxytoccic, an accelerator or as an anæsthetic. However, we applied it the first opportunity we had, and were soon rewarded by finding the painful writhings of the patient much ameliorated, though the contractions were as frequent and as vigorous as previously, The unnecessary painfulness of labor pains had been subdued, the morbid irritability, the hyperæsthesia had been removed, while the contractions were regular, and the os dilating regularly. When a stoppage in the current occurred through battery accidents, the suffering returned at each contraction."

In another case says Dr. Paine: "I found the uterus very inert, and totally insensible to ergot; and after waiting, by the force of circumstance [habit?] twenty-four hours, without a single pain, and having sent for my forceps, I procurred a battery and applied the current to the widely dilated os, when, to my surprise, and as quick as thought, I found the fœtus at the foot of the bed, clear of the mother; the placenta lying loosely in the vagina. The cord being tied and cut, the mother was put to bed; * * * but the surprise was that not one drop of blood stained the bedding or her garments; whereas, if she had been delivered otherwise than by electricity, post partem hemorrhage would have complicated the case."

"My experience, upon the whole is, that electricity is the safest and most natural anæsthetic, and acts without retarding labor in the least; and in many cases, hastening it very much."

With regard to electricity in diseases of children, the Doctor says:

"One of my patients, three months old, had scarcely ceased crying from its birth long enough to nurse. I applied electricity during a terrible spell of crying, and I hardly touched the epigastrium till it was laughing inordinately, for a child. It had only gained two pounds weight in the three months, but in the next eighteen days it gained three pounds, and now, at six months old, is uncommonly healthy, and growing rapidly, and still laughs."

This, certainly, is an exemplification of the force of the adage, "laugh and grow fat;" but we presume the doctor does not mean to tell us that this wonderful effect upon the nutrition and assimilation of the child was produced by that one touch upon the epigastrium? Though he does not say so, it is to be inferred that the child was treated by electricity, subsequent to that one touch. It would be interesting to know how electricity excites the propensity to laugh; we can understand its stimulation of the nutritive process, etc., but—does it tickle? or amuse? or how make a baby laugh? But to resume quotations from Dr. Paine's paper. He says:

"In another paper I have said that all females who menstruate abnormally are the subjects of anæsthesia of the lower half of the person, and also of the mammary glands. Those women procreate crildren as well as healthy women, but do not always have milk; and this anæsthesia of the breasts simply amounts to partial paralysis of function of the gland, and the milk secretion is either imperfect in quantity or quality; for no secreting organ can perform its function healthily that has not a proper nerve support, or is not properly vitalized. The fountain is imperfect, and the product is imperfect and impure, and hence, unfit to nourish the infant dependent on it for sustenance, and the child has to have artificial food, or starve. Full half the babies that are raised by hand and always sick are starvelings in presence of a breast that nobody knows is diseased, because it looks well.

"Instead of prescribing nursery bottles and artificial foods and medicine, I now apply electricity to the mother's breast until sensation is restored, and then it never fails to fill up to running over, and the child gets fat and hearty."

We trust the members of the medical profession will test the doctor's methods, and give the results of their observations. Should Dr. Paine be spared, he is in a fair way to overthrow some of the dogmas of the day with reference to neuro-pathology, and to institute a rational mode of treatment for certain heretofore intractable maladies.

